# The burden of chronic diseases in a rural North Florida sample

**DOI:** 10.1186/1471-2458-13-906

**Published:** 2013-10-01

**Authors:** Henrietta Logan, Yi Guo, Virginia J Dodd, Keith Muller, Joseph Riley

**Affiliations:** 1 Southeast Center for Research to Reduce Disparities in Oral Health, University of Florida, Gainesville, Florida 32610, USA; 2 Health Outcomes and Policy, University of Florida, Gainesville, Florida 32610, USA; 3 Department of Community Dentistry and Behavioral Science, University of Florida, Gainesville, Florida 32610, USA; 4 Department of Health Outcomes and Policy, University of Florida, Gainesville, Florida 32610, USA

**Keywords:** Health disparities, Chronic disease, Rural

## Abstract

**Background:**

The degree of health disparities present in rural communities is of growing concern and is considered "urgent" since rural residents lag behind their urban counterparts in health status. Understanding the prevalence and type of chronic diseases in rural communities is often difficult since Americans living in rural areas are reportedly less likely to have access to quality health care, although there are some exceptions. Data suggest that rural residents are more likely to engage in higher levels of behavioral and health risk-taking than urban residents, and newer evidence suggests that there are differences in health risk behavior within rural subgroups. The objective of this report is to characterize the prevalence of four major and costly chronic diseases (diabetes, cardiovascular disease, cancer, and arthritis) and putative risk factors including depressive symptoms within an understudied rural region of the United States. These four chronic conditions remain among the most common and preventable of health problems across the United States.

**Methods:**

Using survey data (N = 2526), logistic regression models were used to assess the association of the outcome and risk factors adjusting for age, gender, and race.

**Results:**

Key findings are (1) Lower financial security was associated with higher prevalence of cardiovascular disease, arthritis, and diabetes, but not cancer. (2) Higher levels of depressive symptoms were associated with higher prevalence of cardiovascular disease, arthritis, and diabetes. (3) Former or current smoking was associated with higher prevalence of cardiovascular disease and cancer. (4) Blacks reported higher prevalence of diabetes than Whites; Black women were more likely to report diabetes than all other groups; prevalence of diabetes was greater among women with lower education than among women with higher education. (5) Overall, the prevalence of diabetes and arthritis was higher than that reported by Florida and national data.

**Conclusions:**

The findings presented in this paper are derived from one of only a few studies examining patterns of chronic disease among residents of both a rural and lower income geographic region. Overall, the prevalence of these conditions compared to the state and nation as a whole is elevated and calls for increased attention and tailored public health interventions.

## Background

At the national level, there is extensive documentation of past, current, and persistent health inequalities [1] associated with most major chronic diseases among populations differing in socioeconomic status, race, ethnicity, gender, and age [2-5]. National input for Healthy People 2020 recommended expanded health goals and objectives designed to achieve health equity, eliminate disparities, and improve health [6]. Unfortunately, inequalities in health based on geographic region (urban vs. rural) were not reflected in these goals [7,8]. The degree of health disparities present in rural communities and the lack of a rural voice at the policy table has been a growing concern [7,9]. This concern was partially addressed through a contract [Office for Rural Health Policy in the Health Resources and Services Administration of the Department of Health and Human Services and the Southwest Rural Health Research Center] to create a Rural Healthy People 2010 companion to Health People 2010 [7,8]. These excellent documents have given us a picture of the health inequities within and between rural and urban residents and provided health priorities [7].

Many Americans living in rural areas have limited access to quality health care including hospitals [10]. Moreover, 12% of rural Blacks (compared to 10% of rural Whites) reside in a county without a hospital [9]. Rural residents are more likely to engage in higher levels of behavioral and health risk-taking than urban residents, and newer evidence suggests that there are differences in health risk behavior within rural subgroups [11]. Among rural residents, health status is often confounded by factors including education and poverty levels, unemployment and underemployment, being uninsured or underinsured, and geographic distance required to access health care. Rural communities are often characterized by high proportions of minority residents such as Blacks, Hispanics, and Native Americans, as well as levels of poverty exceeding those in urban areas.

One of the challenges faced by health disparities researchers interested in rural health is the lack of reliable baseline data. The dirth of data is partly because of the small number of disease cases in a given geographic area and the cost of surveillance systems [7]. Still, without detailed information about disease patterns and predictors, tailored interventions are next to impossible to design [12,13]. This is especially true in understudied areas such as the rural Southeast where confidentiality requirements of large data sets often limit analyses by sub-populations of rural residents [14].

The objective of this report is to characterize the prevalence of four major and costly chronic diseases [15] (diabetes, cardiovascular disease, cancer, and arthritis) and putative risk factors including depressive symptoms within an understudied rural region of the United States. These four chronic conditions, initially targeted in *Promoting Health/Preventing Disease: Objectives for the Nation*, remain among the most common and preventable of health problems across the United States [15], and three of the four chronic diseases on which we report are included as top priorities for the South [8]. Our rationale for including a measure of depressive symptomology is the emerging importance of depression as a contributor to disease burden and as a public health problem [16,17]. Increasing the understanding of the relationship between depressive disorders and chronic disease by providing reliable baseline data appears vital to public health assessment and the delivery of interventions throughout the rural health care system.

## Methods

### Sample design

The sample targeted a specified geographic region including 36 rural census tracts from Jefferson, Leon, Gadsden, Union, Alachua, and Bradford counties in North Florida, and the survey was conducted from November 2009 through March 2010 [18]. Sampling strata were defined by census block groups according to percent African American, as follows: 30+%, 20–29%, and below 20%. The reported percentages of race for each block group allowed us to select telephone numbers from more densely Black areas for our study. Because geographic location was essential to the overall study and at that time cell phone samples were not available at any geographic unit smaller than the state, only telephones with landlines were included in the sample. This approach also maintained stability for re-contact on a follow-up survey a year later, obtained clearer communication signals, and was optimal for sampling the older population who are more likely to use only landlines. Only people aged 25 or older were included, and a within-household respondent selection procedure was implemented in order to maximize participation of older men and help balance representation by gender. We asked for the oldest male in the household, but allowed immediate substitution (whoever eligible adult was on the phone) if the oldest male was not available. We oversampled Black males based on previous studies reporting lower participation from this community.

### Survey methodology

The University of Florida Bureau of Economic and Business Research Survey Center performed the survey using professional interviewers. Each telephone (landline) number was dialed a maximum of 10 times. A total of 16,000 telephone numbers were dialed, resulting in 2,605 interviews with an average interview length of 21.7 minutes (standard deviation 5.3 minutes). A $15 Wal-Mart gift card was offered for completion of each survey. Duplicate respondents from the same household were eliminated, resulting in a sample of 2,526. A more thorough presentation of the methodology can be found in Riley JL, 3rd, Dodd VJ, Muller KE, et al. [18].

### Description of variables

The survey questions regarding chronic diseases reported in this study were drawn from the Seattle Index of Comorbidity [19]. To allow comparison with other recently reported data, heart attack, congestive heart failure, and stroke were collapsed to define cardiovascular disease (CVD). Data were also collected for the prevalence of diabetes, arthritis, and cancer (excluding skin cancer).

Participants who identified themselves as White or Black were included in the regression analysis, resulting in a sample size of 2,381. Tobacco use was defined as "Never smoker" (not smoking at least 100 cigarettes in their entire life and not smoking daily or on some days), "Former smoker" (smoking at least 100 cigarettes in their entire life and not smoking daily or on some days), or "Current smoker" (smoking at least 100 cigarettes in their entire life and smoking daily or on some days). Data describing use of tobacco other than smoking were excluded from the analysis since less than 4% of respondents reported user experience with smokeless tobacco, spit tobacco, or chewing tobacco. Depression (henceforth referred to as depressive symptoms) was measured through a short form of the Center for Epidemiological Studies Depression Scale (CES-D) (continuous, rescaled to 0–3, with 3 indicating the most depressive symptoms) [20]. A financial security scale (continuous, range 0–2, with 2 indicating the highest) was created based on the following questions: First, participants were asked to describe their financial security: (1) I really can’t make ends meet, (2) I manage to get by, (3) I have enough to manage plus some extra, or (4) Money is not a problem; I can buy about whatever I want. In the second question, the respondents were asked to describe how comfortably they would be able to pay an unexpected $500 medical bill: (1) Able to pay comfortably, (2) Able to pay, but with difficulty, or (3) Not able to pay the bill. Participants with missing answers were excluded, and then a continuous financial security scale was calculated as the weighted average of the two items [21-24]. Education level was classified into six categories: (1) 8th grade or less; (2) some high school, but did not graduate; (3) high school graduate or GED; (4) some college or 2-year degree; (5) 4-year college graduate; and (6) more than a 4-year college degree. Finally, health literacy was measured by three questions designed to gauge a participant’s ability to understand medical information (continuous, range 0–3, with 3 being having the highest health literacy skills) [25]. The questions included "How often do you have a problem understanding the written materials about your medical condition?" "How often do you have a problem understanding what is told to you about your medical condition?" and "How often do you have a problem filling out medical forms by yourself?" The answers to these questions were re-coded to range from 0 to 3, and then a continuous score was calculated as the average of non-missing answers.

### Statistical analysis

To compute sampling weights, the residents were divided into 18 strata defined by three dimensions: census block groups classified by percentage of African American (3 levels: 30 +%, 20–29%, below 20%), gender, and race. Sampling weights were then calculated for the strata using population data for Florida from the 2000 US Census to account for the oversampling of Blacks and men. Differences in demographic variables were evaluated using survey-sample weighted t-tests (for continuous variables) and chi-square tests (for categorical variables). Survey-sample weighted logistic regression models were used to assess the association of the outcome variable (whether a person had a disease) and the risk factors of interest (Tobacco use, Depressive symptoms score, Financial security, Education, and Health literacy score), adjusting for age, gender, and race. Model selection was performed following the strategies described in Muller and Fetterman [26]. A maximum model was constructed with the main effects of the predictors of interest, their interactions with gender and race, and predictor-of-interest × gender × race three-way interactions. Collinearity was evaluated and found acceptable for the maximum model (and hence not a concern for any smaller model). Model selection was based on a backwards step-down selection starting from the interaction terms. A Bonferroni correction controlled for multiple testing with the exclusion P value set at 0.0125 (0.05/4) since there were 4 response variables. Survey procedure PROC SURVEYLOGISTIC of SAS version 9.3 (SAS Institute, Cary, NC) was used for the analysis. We specified stratification and weights using the STRATA and WEIGHT statements.

## Results and discussion

### Description of the study population

Demographics of the study population stratified by race and gender are shown in Table 1. The sample consisted of 2,381 respondents (1,056 men and 1,325 women; 1,676 Whites and 705 Blacks). The mean age was 56.2 years (SD = 15.1 with a range of 25–94 years). Overall, Whites had higher education levels than Blacks (*P* < .0001). Seventeen percent of the Blacks and 7% of the Whites reported education levels of some high school or less. A higher percentage of women had some college training but a higher percentage of men had post-graduate training. Black respondents had a lower financial security score than White respondents (*P* < .0001). In addition, men had a higher financial security score than women (*P* = .0001). Health literacy scores were higher for Whites (p < .0001), whereas no difference was found between men and women (*P* = .1305). Blacks reported higher levels of depressive symptoms than Whites (*P* = .0007); women expressed higher level of depressive symptoms than men (*P* = .0020).

**Table 1:** Demographics by race and gender

**Variable**	**Overall**	**White N = 1676**	**Black N = 705**	***P *value**	**Men N = 1056**	**Women N = 1325**	***P *value**
**Age**	56.2 (15.1)^a^	57.4 (15.4)	53.2 (14.2)	<.0001	56.3 (14.7)	56.1 (15.4)	=.8019
**Education**							
1. 8th grade or less	1 = 2%	1 = 2%	1 = 4%	<.0001	1 = 3%	1 = 2%	=.0001
2. Some HS	2 = 7%	2 = 5%	2 = 13%		2 = 8%	2 = 6%	
3. Completed HS^b^	3 = 27%	3 = 25%	3 = 32%		3 = 28%	3 = 26%	
4. Some college	4 = 30%	4 = 31%	4 = 26%		4 = 25%	4 = 33%	
5. College graduate	5 = 16%	5 = 18%	5 = 11%		5 = 16%	5 = 17%	
6. Post-graduate	6 = 18%	6 = 19%	6 = 14%		6 = 20%	6 = 16%	
**Financial security (range 0–2)**	1.12 (0.59)	1.21 (0.59)	0.89 (0.59)	<.0001	1.17 (0.60)	1.07 (0.58)	=.0001
**Health literacy (range 0–3)**	2.36 (0.73)	2.41 (0.71)	2.24 (0.80)	<.0001	2.34 (0.78)	2.38 (0.69)	=.1305
**Depression (range 0–3)**	0.64 (0.55)	0.62 (0.56)	0.70 (0.51)	=.0007	0.61 (0.52)	0.67 (0.57)	=.0020
**Tobacco use**							
1. Never	1 = 53%	1 = 48%	1 = 67%	<.0001	1 = 45%	1 = 60%	<.0001
2. Former	2 = 30%	2 = 35%	2 = 17%		2 = 36%	2 = 25%	
3. Current	3 = 17%	3 = 17%	3 = 16%		3 = 19%	3 = 16%	

^a^Standard deviations for continuous variables are shown in parentheses.

^b^Completed high school or general educational development (GED).

### Cardiovascular disease

In the communities we sampled, the overall prevalence of CVD was 8.5% (Table 2). Black men had the highest prevalence of CVD (11.7%). The lowest prevalence of CVD was found in White women (5.7%). To increase our understanding of these data we disaggregated CVD to show heart attack, heart failure, and stroke data. The overall prevalence for heart attack was 3.8% (95% CI: 3.0%–4.7%), heart failure 3.9% (95% CI: 2.8%–4.9%) and stroke 3.9% (95% CI: 2.8%–4.9%). Comparison with national data is also shown in Table 3 for heart attack and stroke. (No national data are available for heart failure.)In the regression analysis, tobacco use, depressive symptoms, and financial security were significantly associated with CVD after adjusting for age, gender, and race (Table 4). Former and current tobacco users were more likely than non-users to have CVD with odds ratios of 1.41 (95% CI: 1.02, 1.93) and 1.72 (95% CI: 1.17, 2.53), respectively. Higher levels of depressive symptoms (OR = 1.42, 95% CI: 1.08, 1.85) and lower financial security (OR = 0.49, 95% CI: 0.37, 0.65) were significantly associated with having CVD. Men were more likely to have CVD than women (OR = 2.12, 95%: 1.60, 2.80). There was no difference between Whites and Blacks, nor were there differences by level of education.

**Table 2:** Prevalence of the chronic diseases by gender and race

**Disease**	**Overall**	**Black women**	**Black men**	**White women**	**White men**
**Cardiovascular disease**^**a**^	8.5 (7.1–9.7)^b^	7.9 (5.0–10.3)	11.7 (6.2–14.9)	5.7 (4.2–7.1)	10.2 (8.3–12.0)
Heart Attack	3.8 (3.0–4.7)	1.9 (0.3–3.1)	5.2 (2.1–7.7)	2.1 (1.1–2.9)	5.8 (4.3–7.0)
Heart Failure	3.9 (2.8–4.9)	5.8 (3.0–8.2)	5.2 (1.5–8.1)	2.4 (1.4–3.3)	3.9 (2.5–5.1)
Stroke	3.9 (2.8–4.9)	2.5 (0.7–3.7)	6.2 (2.3–9.1)	3.4 (2.0–4.7)	4.3 (2.4–6.0)
**Diabetes**	14.3 (12.7–15.7)	23.4 (19.7–25.7)	17.1 (12.1–20.1)	11.3 (8.2–13.5)	13.1 (10.8–15.2)
**Arthritis**	27.8 (26.5–28.8)	30.6 (27.7–32.4)	23.9 (19.9–26.2)	30.3 (28.3–31.6)	25.4 (22.1–27.5)
**Cancer**	9.2 (7.8–10.3)	8.8 (5.4–11.5)	7.8 (4.1–10.4)	10.4 (8.2–12.2)	8.0 (6.1–9.9)

^a^A person had at least one of the following conditions: heart attack, heart failure, or stroke.

^b^All prevalence values are percentages (%) age-adjusted to the 2000 US population; 95% confidence intervals are shown in parentheses.

**Table 3:** Prevalence comparison with national data from Centers for Disease Control and Prevention

	**Current sample**	**Florida**	**Nationwide**^**a**^
**Cardiovascular disease**			
Heart Attack	3.8 (3.0–4.7)	5.4 (4.9–5.8)^b^	4.2^b^
Stroke	3.9 (2.8–4.9)	3.5 (3.1–3.9)^b^	2.7^b^
**Diabetes**	14.3 (12.7–15.7)	10.4 (9.8–11.1)^b^	8.7^b^
**Arthritis**	27.8 (26.5–28.8)	27.0 (25.6–28.3)^c^	26.0^c^

^a^Median percentage of all 50 states and District of Columbia.

^b^Data from 2010.

^c^Data from 2009.

All prevalence values are percentages (%) age-adjusted to the 2000 US population; 95% confidence intervals are shown in parentheses.

**Table 4:** Parameter estimates for regression models

	**b**^**a**^	**SD**	**OR**	**95% CI**	***P *value**
**Cardiovascular disease**					
Age	.0577	.0052	1.06	1.05, 1.07	<.0001
Gender (Men)	.7495	.1437	2.12	1.60, 2.80	<.0001
Race (White)	.1066	.1679			.5255
Tobacco use					
Former user	.3401	.1613	1.41	1.02, 1.93	.0350
Current user	.5441	.1959	1.72	1.17, 2.53	.0055
Depression score	.3472	.1360	1.42	1.08, 1.85	.0107
Financial security	-.7080	.1424	0.49	0.37, 0.65	<.0001
**Diabetes**					
Age	.0374	.0040	1.04	1.03, 1.05	<.0001
Gender (Men)	-.5502	.1965			.0051
Race (White)	-.8309	.1661			<.0001
Depression score	.3203	.1147	1.38	1.10, 1.73	.0052
Financial security	-.4134	.1154	0.66	0.53, 0.83	.0003
Education	-.2348	.0686			.0006
Gender x Race	.6703	.2472			.0067
White in males					.4001
White in females			0.44	0.31, 0.60	<.0001
Gender x Education	.2865	.0881			.0011
Education in male					.4121
Education in female			0.79	0.69, 0.90	.0006
**Arthritis**					
Age	.0626	.0040	1.07	1.06, 1.08	<.0001
Gender (Men)	-.3955	.1022	0.67	0.55, 0.82	<.0001
Race (White)	.2292	.1128			.0422
Depression score	.6693	.1030	1.95	1.60, 2.39	<.0001
Financial security	-.3085	.0985	0.74	0.61, 0.89	.0017
Education	-.1470	.0431	0.86	0.79, 0.94	.0006
**Cancer**					
Age	.0538	.0047	1.06	1.05, 1.07	<.0001
Gender (Men)	-.2454	.1342			.0674
Race (White)	.1209	.1539			.4322
Tobacco use					
Former user	.4868	.1482	1.63	1.22, 2.18	.0010
Current user	.6362	.1855	1.89	1.31, 2.72	.0006

*Abbreviations*: CI Confidence interval, OR Odds ratio, SD Standard deviation.

^a^Regression coefficient.

### Diabetes

Overall, 14.3% of the participants self-reported having diabetes (Table 2). A significantly higher proportion of Black women (23.4%) reported having diabetes, while the lowest prevalence of diabetes was found among White women (11.3%). Table 3 shows comparative data to Florida and the nation as a whole. In the regression analysis, higher depression score (OR = 1.38, 95% CI: 1.10, 1.73) and lower financial security (OR = 0.66, 95% CI: 0.53, 0.83) were significantly associated with reporting diabetes (Table 4). Education and diabetes were significantly associated and the strength of the association differed by gender. Among women, participants with a higher education level were less likely to report having diabetes (OR = 0.79, 95% CI: 0.69, 0.90), whereas no relationship between education and diabetes was detected among male respondents. Overall, White women were less likely to report having diabetes than Black women (OR = 0.44, 95% CI: 0.31, 0.60), while there was no difference between White men and Black men. Comparison data to Florida and the nation as a whole are shown in Table 3.

### Arthritis

The overall prevalence of arthritis was 27.8% in our communities (Table 2). The highest prevalence of arthritis (30.6%) was present among Black women; the lowest among Black men (23.9%). As shown in Table 3, the prevalence data for this sample is slightly higher than for Florida and the nation as a whole. A regression analysis (adjusting for age, gender, and race) revealed the presence of arthritis’ significant association with depression score, financial security, and education (Table 4). Participants with a higher depression score were more likely to be arthritic (OR = 1.95, 95% CI: 1.60, 2.39). Participants with a higher education level were less likely to be arthritic (OR = 0.86, 95% CI: 0.79, 0.94). Lower financial security was significantly associated with arthritis (OR = 0.74, 95% CI: 0.61, 0.89). Women were more likely than men to report having arthritis (OR = 0.67, 95% CI: 0.55, 0.82). There were no race differences.

### Cancer

The overall prevalence of cancer (other than skin cancer) was 9.2% in this sample (Table 2). The prevalence of cancer was the highest among White women (10.4%) and the lowest among Black men (7.8%). In the regression analysis, tobacco use was the only significant predictor for cancer after adjusting for age, gender, and race (Table 4). Former tobacco users (OR = 1.63, 95% CI: 1.22, 2.18) and current tobacco users (OR = 1.89, 95% CI: 1.31, 2.72) were more likely to have had a cancer diagnosis than non-users.

### Main findings

Key findings from this study include (1) Lower financial security was associated with cardiovascular disease, arthritis, and diabetes, but not a cancer diagnosis; (2) Depression score (depressive symptoms) was significantly associated with cardiovascular disease, arthritis, and diabetes; (3) Former or current tobacco use was significantly associated with cardiovascular disease and cancer; (4) Blacks reported a higher prevalence of diabetes than Whites. Diabetes was most prevalent among Black women. Women with a higher education level were less likely to report having diabetes than women with lower levels of education; and (5) Overall the prevalence of diabetes and arthritis in these rural North Florida counties was higher than that reported by Florida and national data.

### Characteristics of the communities

Overall, we found that Blacks were less financially secure than Whites. To put these data in perspective, the median household incomes of these rural counties from which the sample was drawn ranged from $33,711 to $44,011. The median household incomes for the State of Florida and the United States are $47,450 and $51,425, respectively [27]. These data point to the economic disadvantage present in the entire rural region from which our sample was drawn. Based on our data, Blacks (especially Black women) reported feeling the least financially secure. This finding is noteworthy because financial security is an important predictor of chronic disease. Individuals who are economically disadvantaged are less likely to have access to resources such as knowledge, money, power, prestige, and beneficial social connections: resources that are important to overall health. Understanding the relationship between access to these resources and chronic disease is integral to resolving health disparities extant in these communities [28]. Interestingly, in our sample, lower financial security accounted for a higher prevalence of chronic disease overall, indicating that regardless of the study context, those with the least financial security are at greatest risk for deleterious outcomes.

Previously, Liao and colleagues [13] reported smoking rates in selected Southeast communities ranging from 12.8% to 30.7% for women and 29.5% to 38.6% for men. Smoking rates in our sample were lower for men (19%) and women (16%).

### Cardiovascular disease

When compared with state data for Florida and national data from the Centers for Disease Control and Prevention (CDC) Behavioral Risk Factor Surveillance System, our targeted rural communities reported a higher prevalence of heart attack and stroke cases. In addition, the prevalence of CVD (having experienced a heart attack, heart failure, or stroke) among Black men in our sample was higher than the prevalence in all the Southeastern communities studied by Liao et al. [13].

### Diabetes

Overall, the diabetes prevalence data collected raises concern. Compared with the CDC data, a significantly higher prevalence of diabetes exists in our targeted rural communities (14.3%, 95% CI: 12.7%–15.7%) than in the state of Florida (10.4%, 95% CI: 9.8%–11.1%) and in the US (8.7%). Compared to findings reported by Liao [13], the prevalence of diabetes among Black women in our sample (23.4%) is significantly higher than that of all the communities sampled (ranged from 10.7% to 20.8%) in that study. Metropolitan areas in this geographic region of Northern Florida have a reported diabetes prevalence of 10.0% (Jacksonville, FL) and 10.9% (Tallahassee, FL), illustrating the impact that financial security and access to care have on this particular rural region [12]. Moreover, 2009 National Health Interview Survey data show that non-Hispanic Blacks (13.2%) were almost twice as likely as non-Hispanic Whites (7.7%) to have been diagnosed with diabetes, which again is far short of the prevalence found in our sample [29]. Nationally, the prevalence of diabetes is somewhat higher in rural than urban areas [30]. By almost any measure, the prevalence of diabetes in our sample is a serious concern warranting local and statewide attention.

### Arthritis

The prevalence of arthritis in our sample (27.8%, 95% CI: 26.5%–28.8%) is slightly higher than that in Florida (27.0%, 95% CI: 25.6%–28.3%) and the US (26.0%). Among this sample, the association between depressive symptoms and arthritis is striking. Our data does not include whether respondents were being treated for depressive symptoms; however, there is extensive data in the literature showing an association between arthritic pain and depressive symptoms [31]. We found no effect of gender or race within depressive symptoms, suggesting that depressive symptoms are playing a role independent of these other factors and should be targeted in interventions tailored for a community of lower socioeconomic status rural individuals.

### Cancer

As a result of advances in earlier detection and treatment, cancer has become curable for some and a chronic disease for others. Thus the data we report is representative of this increasing group of cancer survivors for whom ongoing health services are needed [32]. These individuals often experience late effects of the cancer treatment requiring unanticipated health services [33,34]. Comparison of survivors to the usual cancer prevalence data is somewhat problematic but for our purposes, recent data for adults aged 18 and over in two metropolitan areas in the same geographic region as this sample showed a cancer prevalence of 6.9% (Tallahassee) to 10.9% (Jacksonville) [12]. For one of the counties in our target region, the age-adjusted incidence rate per 100,000 for all cancers was 1,271, which is the highest among all 67 Florida counties [35]. This latter point emphasizes the importance of designing highly effective interventions to meet the needs of cancer survivors for this region [36].

Even though the term cancer includes many different anatomic sites and types, the only significant predictor for cancer diagnosis emerging in our analysis was report of current or former tobacco use. Of note, among cancer survivors in this study, 17.9% report currently smoking (not shown in tables), indicating a need for targeted and effective cessation programs in rural low-income communities for both the healthy (not yet sick) population and cancer survivors [37].

### Depressive symptoms

Depressive symptoms emerged as significant predictors within the cardiovascular disease, arthritis, and diabetes statistical models. This is not to imply causality but it might plausibly be assumed that these results suggest that people with chronic illness are at risk for developing depressive symptoms as a result of dealing with their chronic conditions. Moreover, recent reviews show that depressive symptomology at 12 months post myocardial infarction predicted mortality [38]. Sorting between explanations of causality is not possible with the data at hand. However, since 85% of MHPSAs (Mental Health Provider Shortage Area) are rural, and approximately one third of rural U.S. counties lack any health professionals equipped to address mental health issues [39], it is of concern whether most of those reporting depressive symptoms were receiving care [40].

### Limitations

The results of this study should be interpreted within the context of its design. Study subjects were drawn from 36 rural census tracts from which we oversampled Black males. We believe the sample demographics represent this region of rural Florida and are unique in that the proportion of men to women is similar not only to the region, but to Florida as a whole. Still, we cannot be certain our findings will generalize to rural regions outside of the rural Southeast. However, our purpose was to characterize southern rural communities to enable the design of tailored health-enhancing interventions. Thus we believe this report provides important new information. We also acknowledge that the chronic disease data are based on self-report. The instrument used, however, is similar to that used by other groups including the CDC, making meaningful comparisons possible. In addition, there is potential sampling bias due to the use of random digit dialing of landlines, as the sample may have been more affluent and better educated compared to the overall population [41]. Overall, in spite of the limitations, we believe the strengths of the study prevail and the results merit attention.

### Public health and policy implications

Although policy and financing discussions about rural health often focus on expanding access to clinical services, the public policy agenda could be broadened to include increasing the availability and financing of community based approaches that assist patients in management of their disease. As our findings indicate a high prevalence of diabetes and cardiovascular disease, evidence-based programs that target nutrition, exercise, and smoking cessation may be particularly helpful for rural residents [42,43]. For example, learning to read food labels can increase control over one’s health [44], and this skill could be promoted at the community and population levels. Similarly, recent innovative research in tobacco cessation programs have successfully utilized a voucher system that reinforces behavior change [45,46], and allowing policy makers and community stakeholders to utilize similar methods may aid in decreasing cardiovascular disease. Finally, efforts to reduce the social stigma of seeking and receiving mental health services [47,48] have recently received national-level bipartisan support. However, at the local level, advocates are still needed within communities to encourage individuals with mental health conditions to seek care.

## Conclusions

Health-related disparities are a persistent and serious problem among rural US residents. Large national datasets describing population health are critical for developing and refining the national health objectives present in Healthy People 2020. National data however must be supplemented with local information defining not only chronic diseases and conditions, but behaviors and other pertinent demographic and geographic factors. This paper provides some of that missing data for the Southeastern rural population. Overall, we conclude that the prevalence of these chronic conditions compared to the state and nation data is elevated and increased attention to these rural residents is warranted.

### Human participant protection

This study was approved by the Institutional Review Board at the University of Florida.

## Competing interests

The authors declare that they have no competing interests.

## Authors’ contributions

HL- was responsible for conceptualizing and writing the manuscript. YG and KM - conceptualized the data analysis; conducted and interpreted the data. VD - assisted with writing the manuscript and conceptualized the introduction. JR - designed the study and assisted with writing the manuscript. All authors read and approved the final manuscript.

## Pre-publication history

The pre-publication history for this paper can be accessed here:

http://www.biomedcentral.com/1471-2458/13/906/prepub

## References

[B1] Kawachi I Subramanian SV Almeida-Filho N A glossary for health inequalities J Epidemiol Community Health 2002 56 9 647 652 10.1136/jech.56.9.647 12177079 PMC1732240

[B2] Lutsey PL Pereira MA Bertoni AG Kandula NR Jacobs DR Jr Interactions between race/ethnicity and anthropometry in risk of incident diabetes: the multi-ethnic study of atherosclerosis Am J Epidemiol 2010 172 2 197 204 10.1093/aje/kwq100 20570825 PMC2915488

[B3] Kim D Diez Roux AV Kiefe CI Kawachi I Liu K Do neighborhood socioeconomic deprivation and low social cohesion predict coronary calcification?: the CARDIA study Am J Epidemiol 2010 172 3 288 298 10.1093/aje/kwq098 20610467 PMC2917055

[B4] Kurian AK Cardarelli KM Racial and ethnic differences in cardiovascular disease risk factors: a systematic review Ethn Dis 2007 17 1 143 152 17274224

[B5] Thomas AJ Eberly LE Davey Smith G Neaton JD Stamler J Race/ethnicity, income, major risk factors, and cardiovascular disease mortality Am J Public Health 2005 95 8 1417 1423 10.2105/AJPH.2004.048165 16006418 PMC1449375

[B6] Secretary’s Advisory Committee on National Health Promotion and Disease Prevention Objectives for 2020 Phase I report: Recommendation for the framework and format of healthy people 2020 2008 Washington, DC: Department of Health and Human Resources 91.2

[B7] Bellamy GR Bolin JN Gamm LD Rural healthy people 2010, 2020, and beyond the need goes on Fam Community Health 2011 34 2 182 188 10.1097/FCH.0b013e31820dea1c 21378515

[B8] Gamm LD Hutchison LL Dabney BJ Dorsey AM Rural Healthy People 2010 http://www.srph.tamhsc.edu/centers/rhp2010/

[B9] Elizondo AL Morgan A Crosby RA, Vanderpool RC, Wendel ML, Casey BR History of rural public health in America Rural Populations and Health 2012 San Francisco, CA: John Wiley & Sons 39 50

[B10] Gunderson A Menachemi N Brummel-Smith K Brooks R Physicians who treat the elderly in rural Florida: trends indicating concerns regarding access to care J Rural Health 2006 22 3 224 228 10.1111/j.1748-0361.2006.00036.x 16824166

[B11] Smalley KB Warren JC Klibert J Health risk behaviors in insured and uninsured community health center patients in the rural US South Rural and remote health 2012 12 4 2123 23148502

[B12] Li C Balluz LS Okoro CA Strine TW Lin JS Town M Garvin W Murphy W Bartoli W Valluru B Surveillance of certain health behaviors and conditions among states and selected local areas-Behavioral Risk Factor Surveillance System, United States, 2009 2011 Atlanta, GA: U.S. Department of Health and Human Services Center for Disease Control and Prevention Volume 60 21849967

[B13] Liao Y Bang D Cosgrove S Dulin R Harris Z Taylor A White S Yatabe G Liburd L Giles W Surveillance of health status in minority communities - racial and ethnic approaches to community health across the U.S. (REACH U.S.) risk factor survey, United States, 2009 MMWR Surveill Summ 2011 60 6 1 44 21597458

[B14] Slifkin R Goldsmith L Ricketts T Race and place: Urban–rural difference in health for racial and ethnic minoritieshttp://www.shepscenter.unc.edu/rural/pubs/finding_brief/fb61.pdf23969799

[B15] Centers for Disease Control and Prevention Chronic Diseases and Health Promotionhttp://www.cdc.gov/chronicdisease/overview/index.htm

[B16] Moussavi S Chatterji S Verdes E Tandon A Patel V Ustun B Depression, chronic diseases, and decrements in health: results from the world health surveys Lancet 2007 370 9590 851 858 10.1016/S0140-6736(07)61415-9 17826170

[B17] Chapman DP Perry GS Strine TW The vital link between chronic disease and depressive disorders Preventing chronic disease 2005 2 1 A14 15670467 PMC1323317

[B18] Riley JL 3rd Dodd VJ Muller KE Guo Y Logan HL Psychosocial factors associated with mouth and throat cancer examinations in rural Florida Am J Public Health 2012 102 2 e7 e14 10.2105/AJPH.2011.300504 22390460 PMC3375168

[B19] Fan VS Au D Heagerty P Deyo RA McDonell MB Fihn SD Validation of case-mix measures derived from self-reports of diagnoses and health J Clin Epidemiol 2002 55 4 371 380 10.1016/S0895-4356(01)00493-0 11927205

[B20] Andresen EM Malmgren JA Carter WB Patrick DL Screening for depression in well older adults: evaluation of a short form of the CES-D (center for epidemiologic studies depression scale) Am J Prev Med 1994 10 2 77 84 8037935

[B21] Logan HL Shepperd JA Pomery E Guo Y Muller KE Dodd VJ Riley JL 3rd Increasing screening intentions for oral and pharyngeal cancer Ann Behav Med 2013 46 1 96 106 10.1007/s12160-013-9480-z 23479338 PMC3706731

[B22] Riley JI Pomery E Dodd V Muller K Guo Y Logan H Disparities in knowledge of mouth or throat cancer among rural Floridians Journal or Rural Health 2013 29 3 294 303 10.1111/jrh.12003 23802931 PMC3695415

[B23] Riley JL 3rd Dodd VJ Muller KE Guo Y Logan HL Psychosocial factors associated with mouth or throat exams in rural Florida Am J Public Health 2011 102 2 e7 e14 22390460 10.2105/AJPH.2011.300504 PMC3375168

[B24] Dodd VJ Riley Iii JL Logan HL Developing an oropharyngeal cancer (OPC) knowledge and behaviors survey Am J Health Behav 2012 36 5 589 601 10.5993/AJHB.36.5.2 22584087 PMC3375173

[B25] Chew LD Griffin JM Partin MR Noorbaloochi S Grill JP Snyder A Bradley KA Nugent SM Baines AD Vanryn M Validation of screening questions for limited health literacy in a large VA outpatient population J Gen Intern Med 2008 23 5 561 566 10.1007/s11606-008-0520-5 18335281 PMC2324160

[B26] Muller KE Fetterman BA Regression and ANOVA: An Integrated Approach using SAS Software 2002 Cary, NC: SAS Publishing

[B27] U.S. Census Bureau American Community Surveyhttp://www.census.gov/acs/www/data_documentation/data_main/

[B28] Phelan JC Link BG Tehranifar P Social conditions as fundamental causes of health inequalities: theory, evidence, and policy implications J Health Soc Behav 2010 51 Suppl S28 S40 20943581 10.1177/0022146510383498

[B29] CDC National Health Interview Survey, data Available at http://www.cdc.gov/nchs/nhis.htm

[B30] Dabney B Gosschalk A Gamm LD, Hutchison LL, Dabney BJ, Dorsey AM Diabetes in rural America Rural Health People 2010: a companion document to Health People 2010 1 2003 Texas: College Station 109 116

[B31] Parmelee PA Harralson TL McPherron JA DeCoster J Schumacher HR Pain, Disability, and Depression in 2011 Effects of Race and Sex. J Aging Health: Osteoarthritis 10.1177/0898264311410425 21693669

[B32] Prevention CfDCa Cancer Survivors --- United States 2007 http://www.cdc.gov/mmwr/preview/mmwrhtml/mm6009a1.htm

[B33] Kopp LM Gupta P Pelayo-Katsanis L Wittman B Katsanis E Late effects in adult survivors of pediatric cancer: a guide for the primary care physician Am J Med 2012 125 7 636 641 10.1016/j.amjmed.2012.01.013 22560808

[B34] Schmitz KH Prosnitz RG Schwartz AL Carver JR Prospective surveillance and management of cardiac toxicity and health in breast cancer survivors Cancer 2012 118 8 Suppl 2270 2276 22488701 10.1002/cncr.27462

[B35] Cancer Age-Adjusted Incidence Rates per 100,000 Population 2006 http://statecancerprofiles.cancer.gov/incidencerates/index.php?stateFIPS=12&cancer=047&race=00&type=incd

[B36] Lerro CC Stein KD Smith T Virgo KS A systematic review of large-scale surveys of cancer survivors conducted in North America, 2000–2011 Journal of cancer survivorship : research and practice 2012 6 2 115 145 10.1007/s11764-012-0214-1 22389171

[B37] Gosschalk A Carozza S Gamm LD, Hutchison LL, Dabney BJ, Dorsey AM Cancer in Rural Areas Rural Health People 2010: A companion document to Healthy People 2010, Volume 1 2003 College Station, Texas: Southwest Rural Health Research Center 91 95

[B38] Bekke-Hansen S Trockel M Burg MM Taylor CB Depressive symptom dimensions and cardiac prognosis following myocardial infarction: results from the ENRICHD clinical trial Psychol Med 2012 42 1 51 60 10.1017/S0033291711001000 21682949

[B39] McCord CE Elliott TR Brossart DF Castillo LG Crosby RA, Vanderpool RC, Wendel ML, Casey BR Addressing mental health issues in rural areas Rural Populations and Health 2012 San Francisco, CA: John Wiley & Sons 323 339

[B40] Boecker E Glasser M Nielsen V Weidenbacher-Hoper V Rural older adults’ mental health: satus and challenges in care delivery Rural and remote health 2012 12 2119 1 13 23145784

[B41] Blumberg SJ Luke JV Wireless substitution: Early release of estimates from the National Health Interview Survey July-December 2009 2010 Atlanta, GA: National Center for Health Statistics

[B42] Bopp M Fallon E Community-based interventions to promote increased physical activity: a primer Applied health economics and health policy 2008 6 4 173 187 10.1007/BF03256132 19382818

[B43] Bopp M Fallon EA Bolton DJ Kahl D Engaging community partners to develop a culturally relevant resource guide for physical activity and nutrition Ethn Dis 2012 22 2 231 238 22764648

[B44] Satia JA Galanko JA Neuhouser ML Food nutrition label use is associated with demographic, behavioral, and psychosocial factors and dietary intake among African Americans in North Carolina J Am Diet Assoc 2005 105 3 392 402 discussion 402–393 10.1016/j.jada.2004.12.006 15746826

[B45] Dallery J Raiff BR Contingency management in the 21st century: technological innovations to promote smoking cessation Subst Use Misuse 2011 46 1 10 22 10.3109/10826084.2011.521067 21190402 PMC3085931

[B46] Meredith SE Grabinski MJ Dallery J Internet-based group contingency management to promote abstinence from cigarette smoking: a feasibility study Drug Alcohol Depend 2011 118 1 23 30 10.1016/j.drugalcdep.2011.02.012 21414733 PMC3144260

[B47] Michaels PJ Corrigan PW Measuring mental illness stigma with diminished social desirability effects J Ment Health 2013 22 3 218 226 10.3109/09638237.2012.734652 23323874

[B48] Michaels PJ Corrigan PW Buchholz B Brown J Arthur T Netter C Macdonald-Wilson KL Changing Stigma Through a Consumer-Based Stigma Reduction Program Community Ment Health J 2013 10.1007/s10597-013-9628-0 23760975

